# Disulfiram anti-cancer efficacy without copper overload is enhanced by extracellular H_2_O_2_ generation: antagonism by tetrathiomolybdate

**DOI:** 10.18632/oncotarget.4833

**Published:** 2015-08-07

**Authors:** Ali Calderon-Aparicio, Mary Strasberg-Rieber, Manuel Rieber

**Affiliations:** ^1^ IVIC, Laboratorio de Bioquimica Celular, CMBC, Altos de Pipe, Caracas, Venezuela

**Keywords:** disulfiram, oxidative stress, copper

## Abstract

**Highlights:**

- exogenous SOD increases apoptosis by sub-toxic disulfiram without copper overload
- H_2_O_2_ generation from glucose oxidase also potentiates disulfiram toxicity
- N-acetylcysteine suppresses antitumor potentiation of DSF by H_2_O_2_ generation
- sub-toxic tetrathiomolybdate inhibits potentiation of DSF by SOD

**Background:**

Cu/Zn superoxide dismutases (SODs) like the extracellular SOD3 and cytoplasmic SOD1 regulate cell proliferation by generating hydrogen peroxide (H_2_O_2_). This pro-oxidant inactivates essential cysteine residues in protein tyrosine phosphatases (PTP) helping receptor tyrosine kinase activation by growth factor signaling, and further promoting downstream MEK/ERK linked cell proliferation. Disulfiram (DSF), currently in clinical cancer trials is activated by copper chelation, being potentially capable of diminishing the copper dependent activation of MEK1/2 and SOD1/SOD3 and promoting reactive oxygen species (ROS) toxicity. However, copper (Cu) overload may occur when co-administered with DSF, resulting in toxicity and mutagenicity against normal tissue, through generation of the hydroxyl radical (•OH) by the Fenton reaction.

**Purpose:**

To investigate: a) whether sub-toxic DSF efficacy can be increased without Cu overload against human melanoma cells with unequal BRAF(V600E) mutant status and Her2-overexpressing SKBR3 breast cancer cells, by increasing H_2_O_2_from exogenous SOD; b) to compare the anti-tumor efficacy of DSF with that of another clinically used copper chelator, tetrathiomolybdate (TTM)

**Results:**

a) without copper supplementation, exogenous SOD potentiated sub-toxic DSF toxicity antagonized by sub-toxic TTM or by the anti-oxidant N-acetylcysteine; b) exogenous glucose oxidase, another H_2_O_2_ generator resembled exogenous SOD in potentiating sub-toxic DSF.

**Conclusions:**

potentiation of sub-lethal DSF toxicity by extracellular H_2_O_2_ against the human tumor cell lines investigated, only requires basal Cu and increased ROS production, being unrelated to non-specific or TTM copper chelator sequestration.

**Significance:**

These findings emphasize the relevance of extracellular H_2_O_2_ as a novel mechanism to improve disulfiram anticancer effects minimizing copper toxicity.

## INTRODUCTION

Copper (Cu) is an essential trace element in living systems due to its requirement in a number of enzymes like mitochondrial cytochrome c oxidase, important in fueling cell proliferation [1], copper-zinc dependent SODs required to modulate oxidative stress [2], and copper-activated MAP kinase kinase MEK1 responsible for phosphorylating the mitogen-activated proteín kinase ERK [3]. Basal Cu can help to produce mitogenic reactive oxygen species (ROS) inducing survival and proliferation signaling by moderate levels of hydrogen peroxide (H_2_O_2_) capable of inhibiting redox-sensitive phosphatases which antagonize proteins regulating signal transduction from growth factor and cytokine receptors [4–6]. ROS generation by Cu and H_2_O_2_ can act to promote survival or cell death depending on the extent, persistence and spatiotemporal localization of ROS in specific subcellular compartments inside the cells. Mitogenesis is also controlled through superoxide productionby NADPH oxidases (NOX) enzymes [7] that become activated by recruting Rac1, a small Rho GTPase, critical in promoting malignancy [8, 9]. Rac1-activated NOXs act by transferring electrons from NADPH to molecular oxygen to produce extracellular or intracellular superoxide anion (O_2_^*−^) [11] which cannot cross negatively charged biological membranes. To prevent excessive O_2_^*−^ overproduction and to generate H_2_O_2_-mediated signaling, cells use the copper-dependent SOD1, a cytosolic enzyme that dismutates O_2_^*−^ into H_2_O_2_ (2). However, besides its dismutating activity, SOD1 can also directly regulate NOX-dependent O_2_^*−^ production by binding to Rac1 and inhibiting its GTPase activity [11]. Oxidation of Rac1 by H_2_O_2_ uncoupled SOD1 binding reversibly, producing a self-regulating redox sensor for NOX-generated O_2_^*−^ production [11]. This has led to the suggestion that SOD1 can regulate Nox2-dependent O_2_^*−^ production through its ROS-sensitive control of Rac-GTP hydrolysis. [11]. Targeting NADPH oxidase components to plasma membrane or to other subcellular compartments also helps membrane localization of ROS and activation of downstream redox signaling events [12]. We had a special interest in the plasma membrane NOX [12], a generator of extracellular O_2_^*−^ which may be dismutated to H_2_O_2_ catalytically by extracellular Cu/Zn SOD3, an enzyme that interacts with sulfated glycosaminoglycans which localize this enzyme [13]. In contrast, uncharged extracellular H_2_O_2_ diffuses across membranes in mammalian cells to a limited extent but could readily enter cytoplasm through aquaporin channels [14]. Pharmacologic and genetic inhibition of NADPH oxidase abrogated radiation-induced intracellular O_2_^*−^ generation [15], implying that NADPH oxidase can promote either extracellular or cytosolic production of O_2_^*−^ [14, 15]. Besides the ability of ROS to promote mitogenic signaling that drive aberrant cell proliferation, excessive ROS can lead to DNA damage responses [16–18]. Particularly, in the presence of Cu overload, unprocessed H_2_O_2_ becomes highly toxic because of the generation of hydroxyl radicals (HO), which can damage cells through non-selective oxidation of proteins, lipids, fatty acids, and nucleic acids [19–21]. In humans, several neurodegenerative diseases including Alzheimer's and Parkinson's disease [22, 23] are also characterized by dysregulated copper homeostasis. One of the purposes of this study was to take advantage of the frequent higher levels of mitogenic ROS in cancer cells, to further increase their ROS and promote their preferential cell death [16]. For this purpose, we used disulfiram (DSF), a Cu chelator which has been shown to have an important potential as an anti-cancer agent [24–27]. The DSF molecule, tetraethylthiuram disulphide, decomposes under acidic conditions or upon reduction of its disulphide bridge to yield two diethyldithiocarbamate (DEDTC) molecules [28], which also chelate copper and induce copper-dependent stimulation of ROS [29]. The induction of ROS also occurs by the DSF-mediated chelation of Cu which inhibits SOD1 favouring accumulation of O_2_^*−^ [29]. Tumor cells were reported to respond to Cu deficiency induced by Cu chelators like TTM within the low μM range, by up-regulating the human copper transporter 1 (hCtr1) [30, 31, 33]. Glutathione (GSH) is an abundant physiologic copper chelator and elevated GSH levels enhance hCtr1 expression and transport of copper and platinum [32]. Some mechanisms of acquisition and elimination for Cu are shared by platinum agents like oxaliplatin and cisplatin [33, 34], which function as competitors for hCtr1-mediated copper transport, resulting in reduced cellular copper levels [33, 34]. Since extracellular superoxide anions and H_2_O_2_ have been implicated in stimulation of proliferation [35–37], this report investigated whether exogenous addition of SOD [35] or glucose oxidase [38, 39] a source of limited amounts of H_2_O_2_ [38] augment the anti-tumor efficacy of sub-toxic DSF without increasing Cu. This was based on our earlier demonstration that H_2_O_2_ plays an important role in Cu[DEDTC]_2_ cytotoxicity, since the latter is counteracted by exogenous peroxidase activity [29].

## RESULTS

### Exogenous SOD promotes sublethal DSF toxicity antagonized by thiomolybdate or N-acetylcysteine in human melanoma cell lines irrespective of V600E-BRAF status

**Figure 1:**
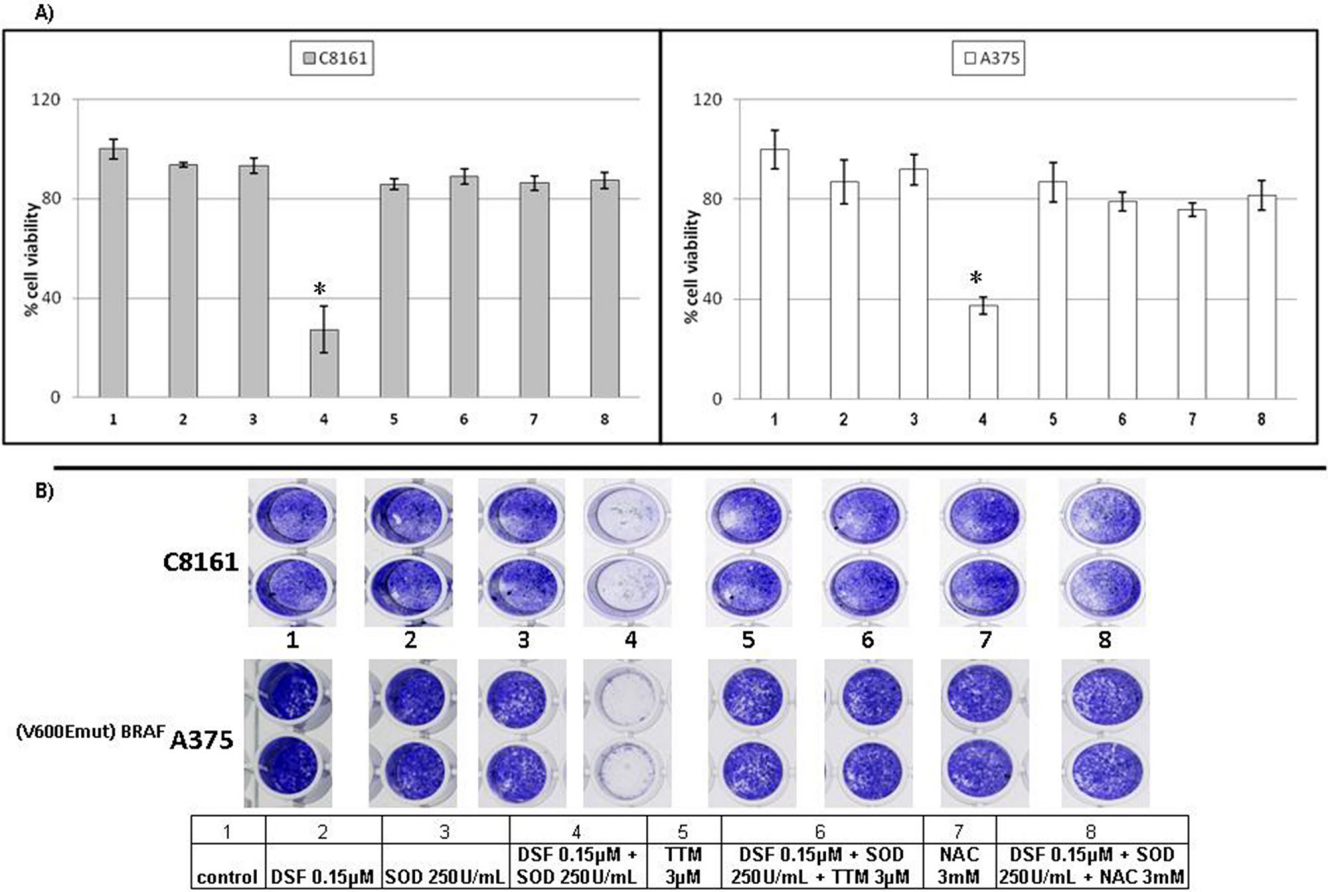
SOD promotes sublethal DSF toxicity antagonized by thiomolybdate or N-acetylcysteine irrespective of *BRAF* status **A.** Changes in viability were estimated in sub-confluent cells seeded overnight followed by exposure to the treatments indicated for 72 hours in 96 well plates (*n* = 8), using the Alamar Blue resazurin/resorufin assay described under Methods **B.** Differences in cell survival were assayed after the indicated treatments for 72 hours by fixing cells with 70% ethanol and staining with crystal violet, as described under Methods.

Since plasma membrane NOX activity can produce extracellular superoxide anions important in cell survival [35–37], exogenous SOD was tested for its ability to potentiate sub-toxic concentrations of DSF. Neither 0.15 μM DSF nor 250 units/ml of exogenous SOD exerted significant toxicity against C8161 melanoma or V600E-BRAF mutant A375 melanoma. However, joint treatment with both agents significantly killed both cell types. Addition of 3 μM TTM, another Cu chelator [30, 45] or the glutathione precursor N-acetylcysteine (NAC) [32] reversed the toxicity induced by SOD and 0.15 μM DSF, in contrast with the lack of toxicity of TTM as a single agent (Figure 1A & 1B).

### Apoptosis-associated PARP cleavage is increased by DSF and SOD and antagonized by copper chelator TTM

**Figure 2:**
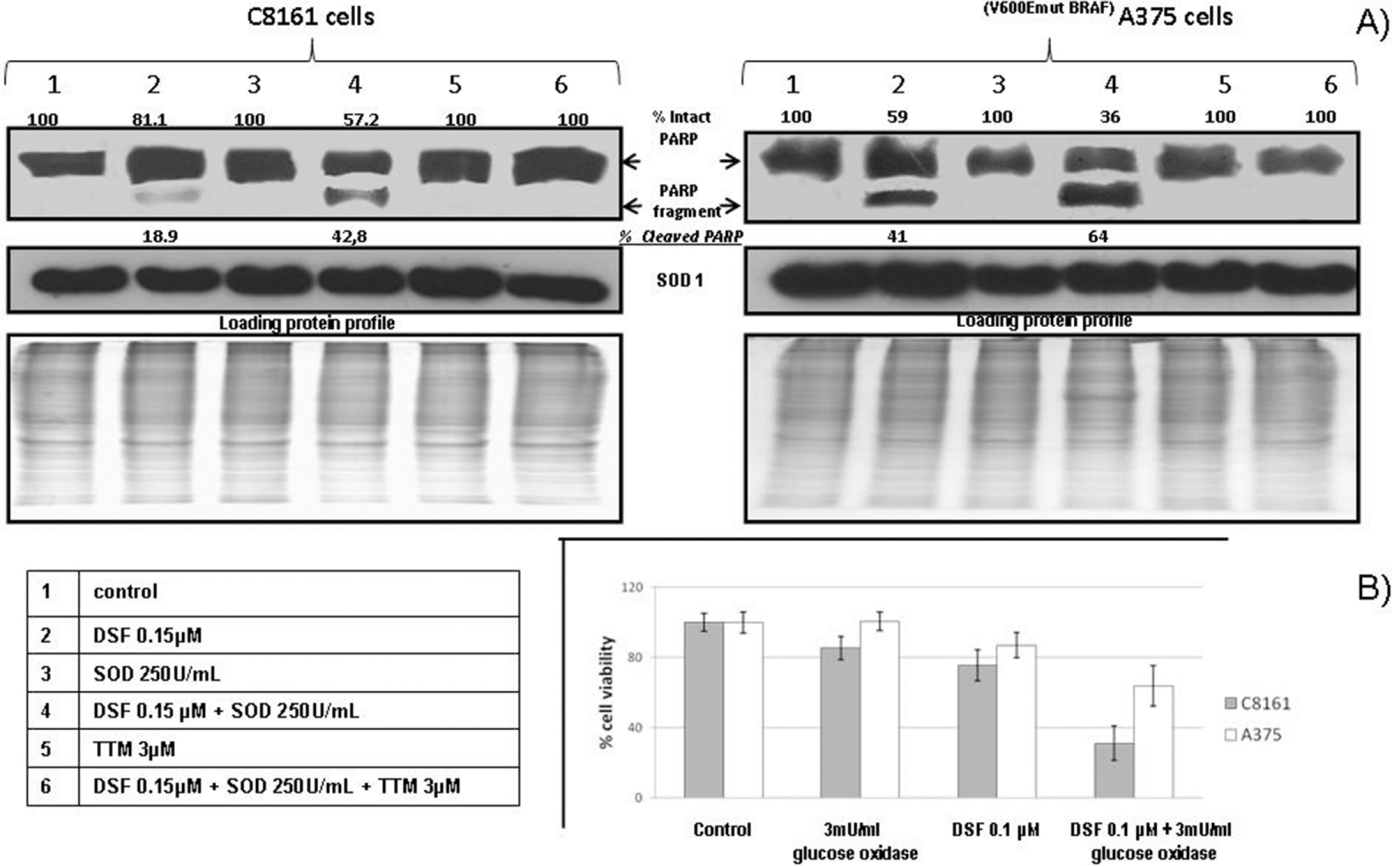
**A.** Apoptosis-associated PARP cleavage induced by DSF and SOD is antagonized by copper chelator TTM in human melanoma cell lines Cells were seeded in 5 cm tissue culture dishes overnight, followed by exposure to the indicated treatments for 30 hours, and harvesting of adherent and floating cells for SDS-PAGE electrophoresis, Western blot analysis and PARP fragmentation detection by chemiluminescence, as described under Methods. **B.** Glucose oxidase enhances DSF toxicity preferentially in C8161 cells Changes in viability were estimated in sub-confluent cells seeded overnight in octuplicates followed by exposure to the treatments indicated in 96 well plates (*n* = 3), using the Alamar Blue resazurin/resorufin assay described under Methods

To find out whether the potentiation of sub-toxic DSF by exogenous SOD involved apoptosis-associated PARP cleavage [29], we used immune blotting. This revealed partial PARP cleavage in cells singly exposed to DSF. However, the ratio of cleaved to intact PARP was increased when cells were jointly treated with SOD and DSF. In both cell types irrespective of their BRAF status, PARP cleavage was reversed by 3 μM TTM (Figure 2A).

### Glucose oxidase enhances DSF toxicity preferentially in C8161 cells

Since exogenous SOD enhancement of sub-toxic DSF mediated cell death (Figure 1) is likely to involve dismutation-mediated H_2_O_2_ generation, we also used exogenous glucose oxidase, another H_2_O_2_ generator [38, 39]. This revealed no toxicity by DSF or glucose oxidase at the concentrations indicated when used as single agents. However, their joint addition significantly increased melanoma cell death, partly attenuated in the BRAF-mutant A375 cells (Figure 2B).

### Toxicity of lethal DSF concentrations is antagonized by higher sub-toxic TTM levels in melanoma cell lines

**Figure 3:**
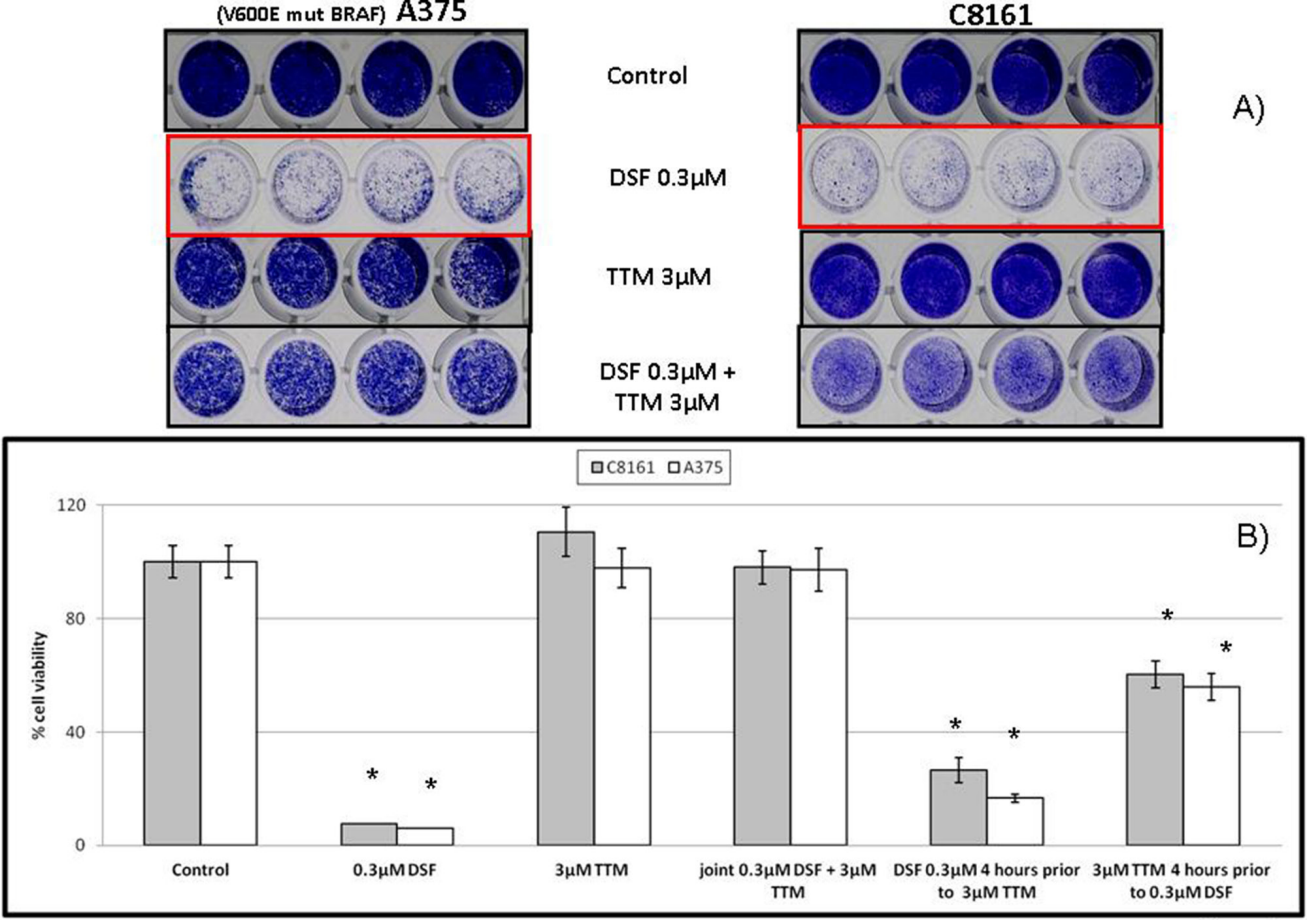
**A.** Toxicity of lethal DSF concentrations is antagonized by sub-toxic TTM levels Sub-confluent cells seeded overnight in octuplicates were exposed to the treatments indicated for 72 hours in 96 well plates (*n* = 3). Differences in cell survival were assayed after the indicated treatments for 72 hours by fixing cells with 70% ethanol and staining with crystal violet, as described under Methods. **B.** Inhibition of lethal disulfiram (DSF) toxicity by tetrathiomolybdate (TTM) requires joint addition C8161 and A375 melanoma cells were seeded at sub-confluency in 96-well plates and allowed to adhere for 24 hr. Cell cultures in octuplicates were then treated with TTM o DSF whenever indicated for 4 hr. Cultures were then washed and and treated as indicated for further 72 hr. Cell viability was then measured fluorometrically with Alamar Blue. Results shown are representative of 3 different assays with *n* = 3 in each experiment.

When co-administered with Cu, both DSF [30, 43] and TTM [45] have been used as anti-cancer agents. Since Figures 1&2 showed that sub-toxic 0.15 μM DSF potentiation by exogenous SOD is reverted by 3 μM TTM, we also investigated whether TTM reverted cell death induced by toxic 0.3 M DSF in the absence of exogenous SOD. This confirmed that TTM without copper supplementation above that pre-existing in culture medium and serum supplementation is not toxic as a single agent up to 5 μM against C8161 or A375 cells. In contrast, 0.3 μM DSF toxicity was counteracted by 3 or 5 μM TTM, which by itself was toxic without Cu co-administration at ≥ 10 μM, compared to controls (Figure 3A).

### Inhibition of lethal disulfiram (DSF) toxicity by tetrathiomolybdate (TTM) requires joint addition

Since both TTM and DSF are copper chelators but the above results showed that 3 μM TTM protected from DSF toxicity, we asked whether delayed addition of DSF or TTM influenced their biological behavior in the absence of Cu co-administration. When 0.3 μM DSF was added 3 hours prior to addition of a 10 fold TTM molar excess, attenuation of DSF toxicity by TTM was significantly diminished and this was partly modified when TTM was added 4 hours before DSF. However, joint addition of DSF and TTM completely reverted DSF toxicity, even in BRAF-mutant A375 cells (Figure 3B).

### SOD augmentation of cell death and PARP cleavage by sub-toxic DSF in Her2-overexpressing SKBR3 breast carcinoma

**Figure 4:**
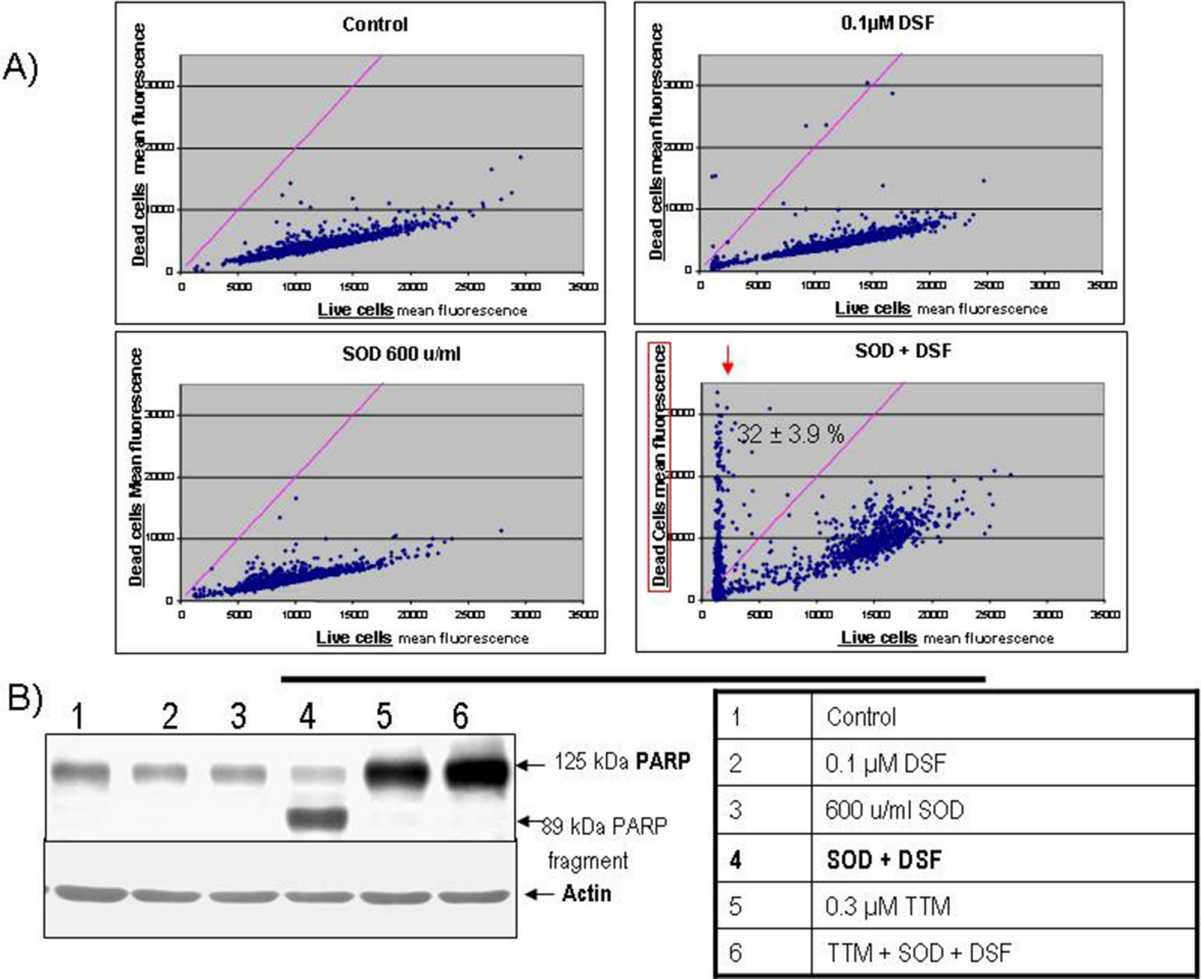
SOD augmentation of sub-toxic DSF-mediated cell death in Her2-overexpressing SKBR3 breast carcinoma **A.** Live-dead cytofluorometric analysis showing an increase in dead cells in sub-confluent cultures exposed for 20 hours to DSF and SOD. **B.** Apoptosis-associated PARP cleavage assayed by immune blot from extracts from sub-confluent cultures exposed for 20 hours to the indicated treatments.

We investigated whether exogenous SOD also enhanced the efficacy of sub-toxic DSF against Her2-overexpressing SKBR3 breast carcinoma which harbour a mutant p53 R175H [29, 41]. Cytofluorometric live-dead analysis indicated a majority of live cells in control cultures or in those singly treated with DSF or SOD. However, SOD cooperated with DSF to increase the dead cell population to about 30% (Figure 4A). Apoptosis-associated PARP cleavage normalized to actin levels was used to extend the live-dead studies, confirming PARP fragmentation only in SKBR3 cells exposed to DSF+SOD, effect inhibited by concomittant TTM addition (Figure 4B) extending the results shown in Figure 2 for human melanoma cell lines.

## DISCUSSION

**Figure 5:**
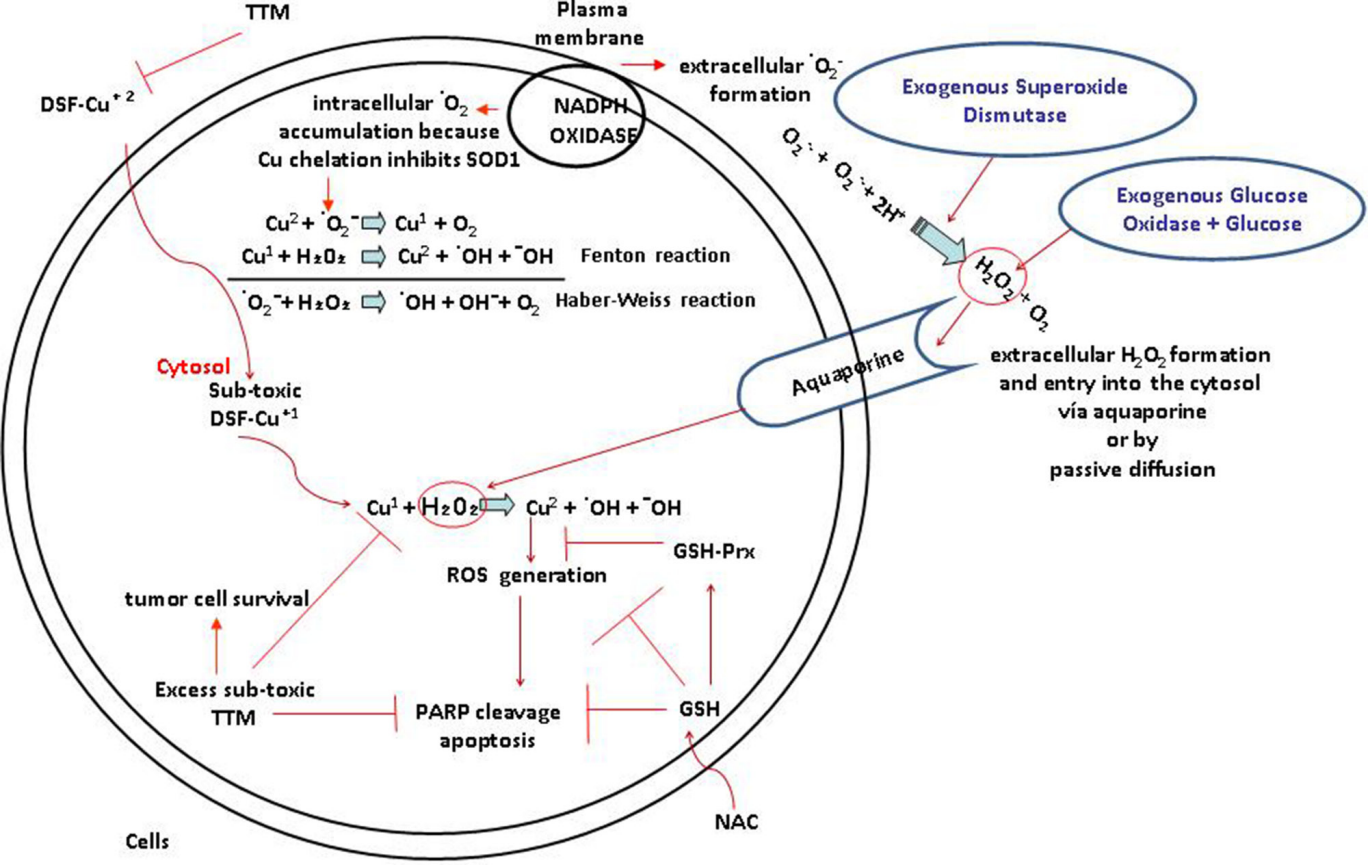
Summary. Toxicity induced by sublethal levels of DSF and low copper is increased by extracellular H_2_O_2_ and counteracted by TTM or NAC In the absence of Cu co-administration, sub-toxic DSF decreases basal Cu and inhibits Cu-dependent cytosolic SOD1 which cannot generate cytosolic H_2_O_2_ [35]. Plasma membrane NADPH oxidase increased in tumor cells favours extracellular superoxide-mediated H_2_O_2_ formation [36] which potentiates sub-toxic DSF-Cu-regulated Fenton-Haber Weiss redox reactions. Lower catalase in melanoma vs melanocytes [50] and exogenous SOD [35] contribute to preservation of extracellular superoxide-mediated H_2_O_2_ formation. This H_2_O_2_ can enter the cells via aquoporins [14] to inhibit Tyrosine PTPases [35] favouring receptor tyrosine kinase activation mitogenic signaling. However, the Cu bound to DSF restricts Cu available to activate MEK1, promoting cell death because Cu and H_2_O_2_ driven mitogenic signaling [3] require Cu for -MEK1-driven ERK activation [43] but this Cu is restricted through chelation by DSF. DSF-Cu toxicity linked to high ROS [44, 47, 53, 54] is inhibited by non-toxic levels of TTM which sequesters Cu from DSF reverting toxicity and apoptosis-associated PARP cleavage. N-acetylcysteine (NAC), a precursor of glutathione which also chelates Cu [34], also antagonizes the potentiation of sub-toxic DSF by SOD.

**Figure 6:**
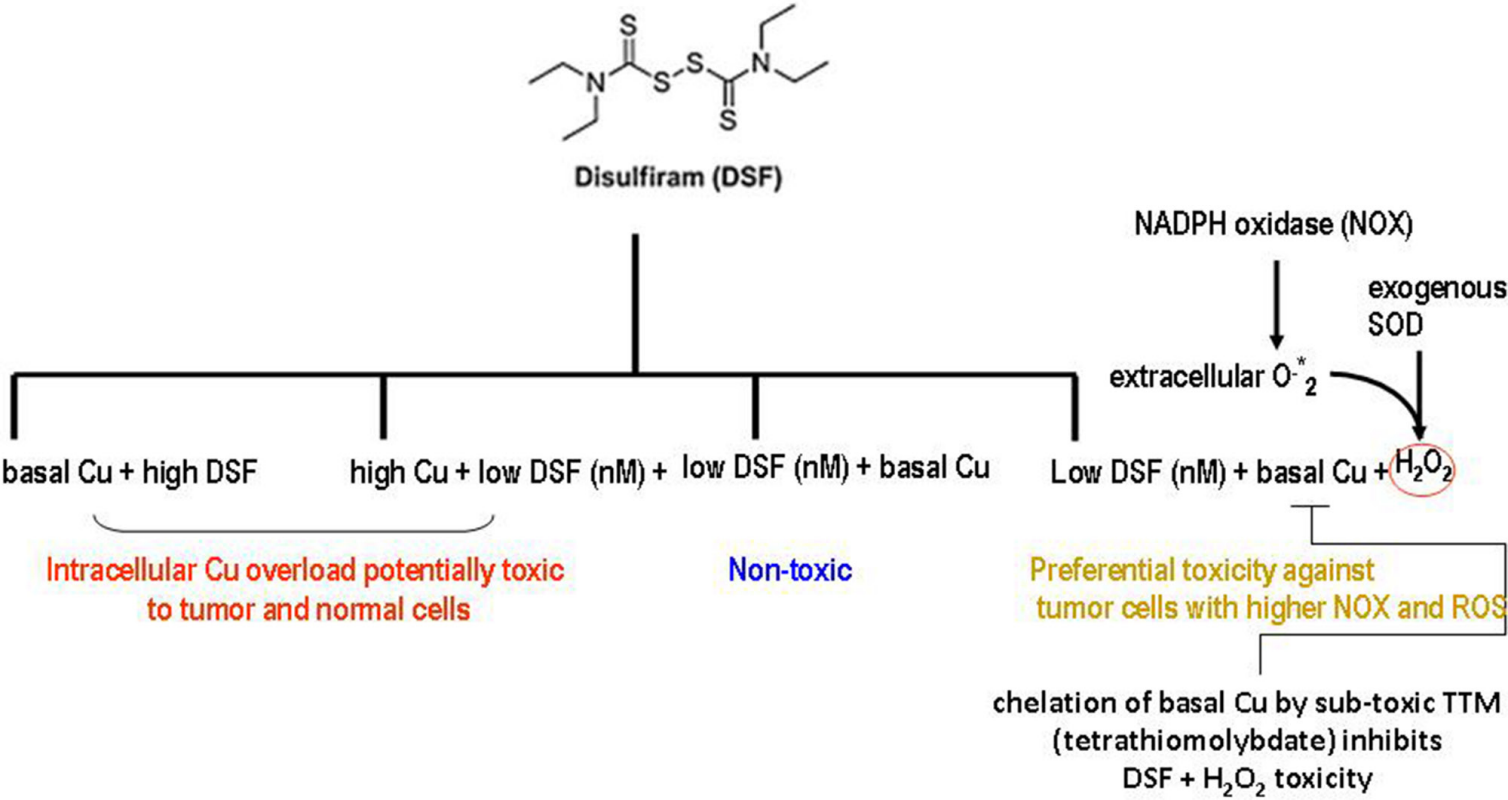
SummarySelective DSF anti-tumor activity requires basal Cu bound to sub-toxic DSF rather than TTM-Cu chelation or extracellular H_2_O_2_ Sub-toxic DSF is more likely to kill preferentially tumor cells with higher basal Cu levels and greater NOX and ROS activity.

New strategies for selectively killing cancer cells are required to: a) diminish toxicity against normal tissue; b) inhibit growth-promoting features preferred by malignant cells. Increased production of growth-promoting ROS [16, 21, 35] and higher Cu levels [1, 43], frequently seen in cancer cells relative to normal cells, may be selectively used to promote tumor cell death [1]. DSF, in ongoing clinical trials is a copper chelator with preferential toxicity against cancer cells [3, 37, 39] via ROS overproduction when co-administered with Cu [24–27, 44]. However, one of the caveats limiting therapeutic DSF use with Cu is its significant toxicity against normal tissue [22, 23]. In the gastric tract or in an acidic tumor environment, DSF is promptly metabolized to DEDTC [28] which also chelates Cu(II). This DEDTC-Cu complex is more stable than DSF itself, thereby facilitating anticancer activity. DSF co-administration with Cu highly increases ROS partly by the Fenton reaction of Cu with H_2_O_2_ generating the •OH hydroxyl radical [19]. Although physiological extracellular levels of transition metals like Fe^2+^ or Cu^1+^ can catalyze a •OH-generating Fenton reaction outside the cell, the fact that •OH is about 10^9^ times less stable compared to H_2_O_2_ [44] and its ability to react with extracellular proteins and lipids [44] or platelets [45], makes improbable that it will reach sensitive intracellular tumor targets, unlike the •OH produced in an intracellular Fenton reaction. Previously, we showed an involvement of H_2_O_2_ in Cu[DEDTC]_2_ cytotoxicity, since the latter was counteracted by exogenous peroxidase activity or thiol anti-oxidants like NAC [29]. As a follow-up, this report is the first to show that without Cu overload, exogenous SOD potentiates sub-toxic DSF increasing cell death in two wt p53 human melanoma cell lines differing in their V600E-mutant BRAF status and in mutant p53 R175H SKBR3 breast carcinoma cells overexpressing the EGFR2/Her2 oncogene. No comparable toxicity was evident when these agents were used individually. In these studies, exogenous superoxide dismutase (SOD), but not heat-inactivated SOD promoted DSF sub-lethal toxicity (not shown), implying its dismutating activity as an extracellular H_2_O_2_ generator. This potentiation of sub-toxic DSF was also seen with glucose oxidase, another H_2_O_2_ generator [38, 39] and was prevented by sub-toxic 3 μM of Cu chelators like TTM or bathocuproine (not shown) or by NAC, a glutathione (GSH) precursor. Sub-toxic levels of the Cu chelator DSF or exogenous SOD [35] are likely to cooperate to increase Cu and H_2_O_2_ favouring their participation in an intracellular Fenton reaction (Figure 5, summary). Physiologically, the plasma membrane localized NADPH oxidase transfers electrons from NADPH to molecular oxygen to produce extracellular superoxide anion which can be processed by SOD to generate extracellular H_2_O_2_. At sub-toxic levels, H_2_O_2_ increases ROS which drive the Ras/BRAF/mitogen-activated protein kinase ERK signaling, in which Cu influx by its hCtr1 transporter is required. In the absence of physiological Cu, cells may die because MEK activation of the ERK1/2 survival pathway requires Cu supplementation [3, 30, 43]. However, cells survive co-treatment of DSF and exogenous SOD when TTM or the glutathione precursor NAC are added. A possible reason for the attenuation of cell death by TTM may be the high depletion of Cu caused by TTM. This is likely to diminish available Cu [31] triggering a Cu homeostasis response through activating the function of the Cu transporter hCtr1 also used by platinum [32, 33], to increase Cu or cisplatin transport into tumor cells. The anti-oxidant NAC also protects from ROS induction by DSF, up-regulating glutathione, another inducer of hCtr1 [34]. Facilitation of Cu entry by hCtr1 would reactivate Cu-dependent MEK survival signaling and cancer cell proliferation [3, 44]. TTM, previously known as an anti-cancer Cu chelator when used as a single agent [46] paradoxically also helped revert apoptosis-associated PARP cleavage mediated by DSF and SOD, implying that basal Cu sequestration by TTM from DSF diminishes the latter ROS-inducing ability (47), implying that Cu bound to TTM behaves very differently to Cu bound to DSF. A recent comparison of Cu chelators TTM and penicillamine, showed that only the latter increased available Cu and oxidative stress in mouse brain, while TTM administration did not lead to comparable results [48]. Together, these data suggest that the potentiation of sub-toxic DSF activity against human melanoma and breast carcinoma cells irrespective of their BRAF or p53 mutant status and EGFR2/HER2 over-expression, is not merely related to Cu sequestration or increased Cu uptake by Cu chelators or ionophores, but rather to the ability of low DSF levels to increase basal Cu-dependent generation of ROS through higher intracellular hydroxyl radicals (•OH) production [19], since it is attenuated by the Cu chelator TTM or by the anti-oxidant NAC (Summary, Figure 6).

Our findings that increasing H_2_O_2_ generation potentiates DSF toxicity is compatible with others indicating that DSF administration *in vivo* potentiates hyperoxia-mediated oxidative toxicity, via conversion to diethyldithiocarbamate and SOD inhibition [49]. Although it was frequently argued that DSF toxicity was mostly dependent on a stable DSF complex with Cu, others recently showed that upon addition of the Cu (II) ions to the media, the cells are exposed to a rapid redox decomposition of DSF with a catastrophic release of H_2_O_2_, implying generation of the latter as crucial in DSF toxicity [50]. The translational potential of enhancing DSF toxicity by gradual H_2_O_2_ delivery to tumor cells [38, 39] by small micron-sized glucose oxidase –microspheres as a source of exogenous ROS [51], but avoiding toxicity from Cu overload is supported by data showing that melanoma cells have increased SOD which generates more H_2_O_2_ but lower catalase activities, compared to normal human melanocytes [52]. This study emphasizing the relevance of exogenous H_2_O_2_ generation by exogenous dismutase activity like that provided by extracellular SOD3 [13] is compatible with other results showing that loss of SOD3 expression promotes an invasive phenotype in human pancreatic adenocarcinoma [53].

Significance.- In the absence of Cu co-administration with DSF which lowers non-specific toxicity [22, 23], basal Cu in sera is effectively chelated and delivered to cells by ceruloplasmin. Although the Cu to ceruloplasmin ratio is not significantly altered, Cu and ceruloplasmin levels are increased significantly in the cancer patients compared to controls [54]. Since basal Cu [1, 54] and intrinsic ROS levels [55, 56] are higher in some cancer cells [30], use of sub-toxic DSF with H_2_O_2_ generators represents a potentially new approach to selectively target cancer cells, limiting the toxic side effects associated with Cu overload against normal cells (Summary, Figure 6).

Considering that Her2-overexpressing SKBR3 cells and V600E-BRAF-mutant A375 melanoma cells respond to sub-toxic DSF and H_2_O_2_ generators, it may be wortwhile to further investigate pre-clinically whether DSF cooperates to perturb redox homeostasis and attenuate resistance to targeted therapy with trastuzumab in Her2-overexpressing breast cancer [57] or with vemurafenib in V600E-mutant BRAF melanoma cells [58].

Our rationale for DSF treatment avoiding Cu overload while augmenting intracellular H_2_O_2_ generation, is to target tumor cell populations with higher ROS and Cu levels [54, 55]. Basal Cu rather than Cu supplementation with DSF, is likely to preferentially restrict cancer cell proliferation and survival, because of their greater copper requirement [1, 3, 30, 47].

## MATERIALS AND METHODS

### Human cell cultures

- C8161 melanoma cells have been reported to lack the BRAF^V600E^ mutation [40] (http://www.wistar.org/lab/meenhard-herlyn-dvm-dsc/page/mapk-and-pi3k-pathways)(https://cansar.icr.ac.uk/cansar/cell-lines/C81-61/0) and show greater resistance to MEK inhibition in three-dimensional culture [36].
- A375 melanoma cells are BRAF V600E-mutant [41]. The BRAF^V600E^ kinase activating mutation is found in more than 60% of melanomas and promotes MAPK pathway signaling independent of other mutations [41].
- SKBR3 human breast carcinoma cells originated from mammary gland were derived from a metastatic site. These cells harbour a p53R175H mutation and over-express the EGF receptor 2/Her2 oncogene [42].

All cells used in this study were maintained in Dulbecco's medium supplemented with 20 mM glucose, 4 mM glutamine and 10% fetal bovine serum unless otherwise indicated.

### Relative cell viability/ metabolic activity

This was estimated with Alamar Blue (resazurin) obtained from Life Technologies (Carlsbad, CA). It measures intracellular redox mitochondrial activity by quantitating the cell-catalyzed conversion of non-fluorescent resazurin to fluorescent resorufin. For these experiments, cells (6 × 10^3^) were allowed to adhere overnight in 96 well TC microtiter dishes. After the corresponding treatments, Alamar Blue was added to 10% of the cell volume without removing medium containing poorly adherent or dead cells [42], and fluorescence was measured 4 h later in a Labsystems Fluoroskan Ascent microplate reader at an excitation of 544 nm and an emission of 590 nm [29, 42]. Changes in cell viability relative to controls was measured after 48–72 hours treatment, in an end-point fluorometric resazurin reduction method assay. The results from a representative experiment are shown, expressed as relative fluorescence ± SD.

### Live-dead assays

Live-dead ratio was determined by adding Calcein AM and propidium iodide directly to sub-confluent cultures containing approximately 5–7 × 10^3^ adherent cells. Calcein acetoxymethyl (AM) is a membrane-permeable live-cell labeling dye. Upon entering the cell, intracellular esterases cleave the AM ester group, yielding the membrane-impermeable Calcein fluorescent dye, optimally excited with a 488 nm laser at 495 nm with a peak emission of 515 nm. Dead cells with compromised cell membranes do not retain Calcein but can be identified by uptake of the non-permeable propidium iodide which preferentially stains DNA, detected with the same laser at ≥ 605 nm. Cytofluorometry was used to determine the relative ratio of live cells with green fluorescence and dead cells with red fluorescence in an Isocyte laser spectrofluorometer, without washing away dead cells or removing the fluorochromes.

### Western blot analysis

Sub-confluent cells were harvested in PBS containing protease and phosphatase inhibitors using a rubber policeman. Extracts were prepared in cell lysis buffer (50 mM Tris–HCl, pH 8, 120 mM NaCl, 50 mM NaF, 0.1 mM sodium vanadate, 5 mM EDTA, 10 μg/ml each of leupeptin, soybean trypsin inhibitor, and aprotinin, 1 mM phenylmethylsulfonyl fluoride, 0.4% Nonidet P40). Seventy-five micrograms of protein was loaded into each well of a 11% SDS-polyacrylamide gel and electrophoretically separated. After protein transfer, the membranes were blocked with TBS (Tris-buffered saline, pH 7.5) containing 0.1% Tween-20 and 5% nonfat skim milk. All the chemicals above indicated were obtained from Sigma–Aldrich (St. Louis, MO, USA). Antibody detecting both the intact and apoptosis-mediated cleaved PARP forms [29, 41] and antibody versus SOD1 were from Cell Signaling (Waltham, MA, USA).

### Crystal violet staining of surviving adherent cells

Cells were subjected to the treatments indicated in each case. Subsequently, the unattached dead population was removed after washing twice in isotonic phosphate-buffered saline. Surviving cells were evidenced following fixation in 90% ethanol and cell staining with 0.5% crystal violet in 30% ethanol (both from Sigma–Aldrich, St. Louis, MO, USA) [41].

### Statistical studies

Standard deviations (S.D.) were used to determine a statistically significant difference in the median values shown for metabolic activity/cell viability and similar assays. These were repeated at least 2 times. Generally, S.D. results usually were within ± 5% with a 95% statistical significance. The criterion for statistical significance was taken as *p* < 0.05 by Student's *t* test, whenever indicated by *.

## Abbreviations

DSF: Disulfiram
TTM: tetrathiomolybdate
Cu: copper
SOD: Superoxide Dismutase
(NOX): NADPH-oxidase
